# Supplementary material for: KegAlign: Optimizing pairwise alignments with diagonal partitioning
Source: bioRxiv. 2024 Sep 3:2024.09.02.610839. Preprint. [Version 1] doi: 10.1101/2024.09.02.610839 (PMC11398343; doi:10.1101/2024.09.02.610839)
Supplement: Supplement 1 [file NIHPP2024.09.02.610839v1-supplement-1.pdf]

bioRxiv preprint doi: https://doi.org/10.1101/2024.09.02.610839; this version posted September 3, 2024. The copyright holder for this preprint (which was not certified by peer review) is the author/funder, who has granted bioRxiv a license to display the preprint in perpetuity. It is made available under aCC-BY 4.0 International license.

Supplemental Figures

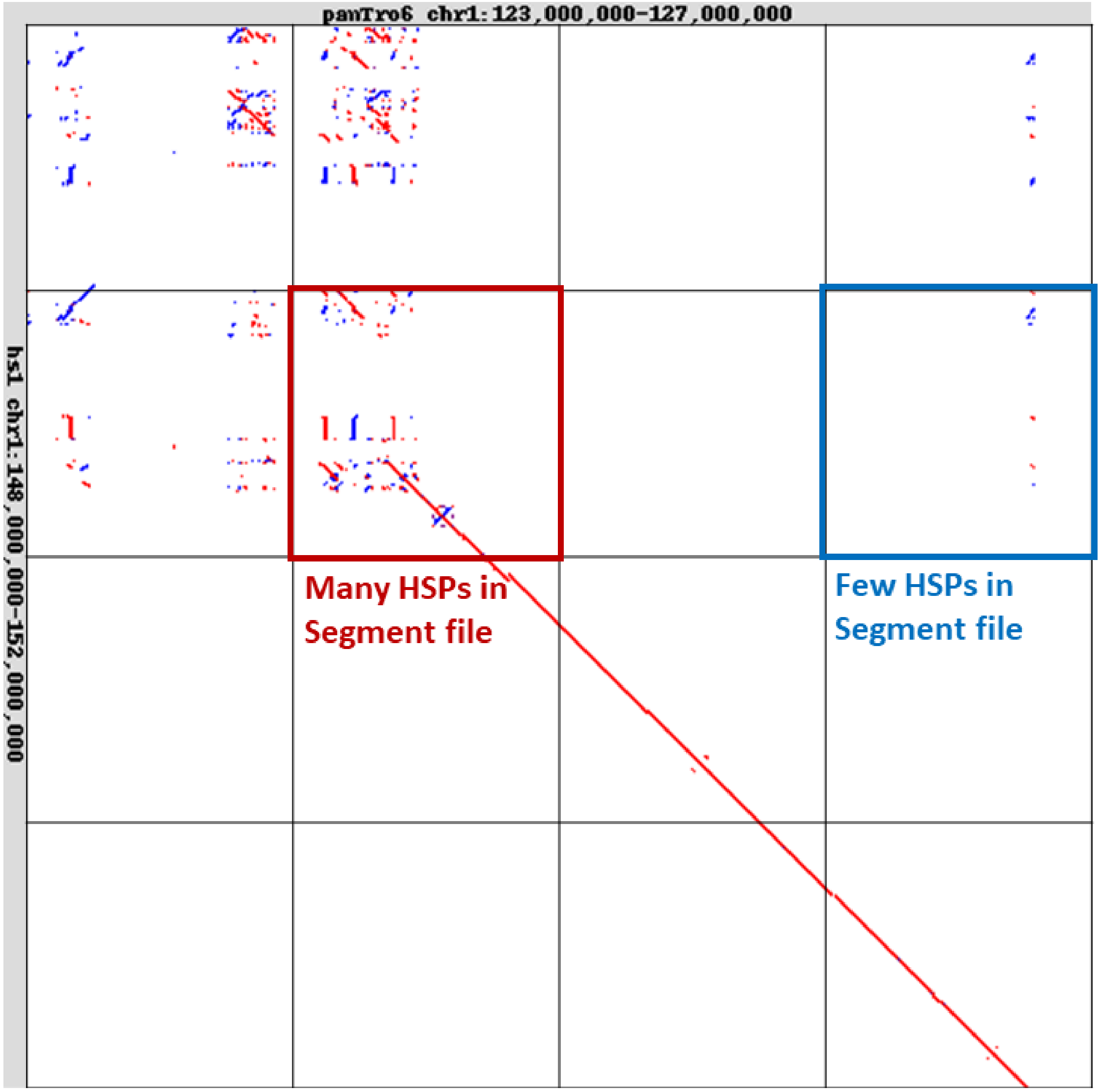

Supplement Figure 1. Human – Primate chr1 alignment. Each chromosome has ~250 million nucleotides. Each segment file contains HSPs from a single chunk. Each box indicates a 10x10Mbp chunk. Some chunks can have very few alignments as shown in the blue box, while others can have significantly more, as shown in the red box.

bioRxiv preprint doi: https://doi.org/10.1101/2024.09.02.610839; this version posted September 3, 2024. The copyright holder for this preprint (which was not certified by peer review) is the author/funder, who has granted bioRxiv a license to display the preprint in perpetuity. It is made available under aCC-BY 4.0 International license.

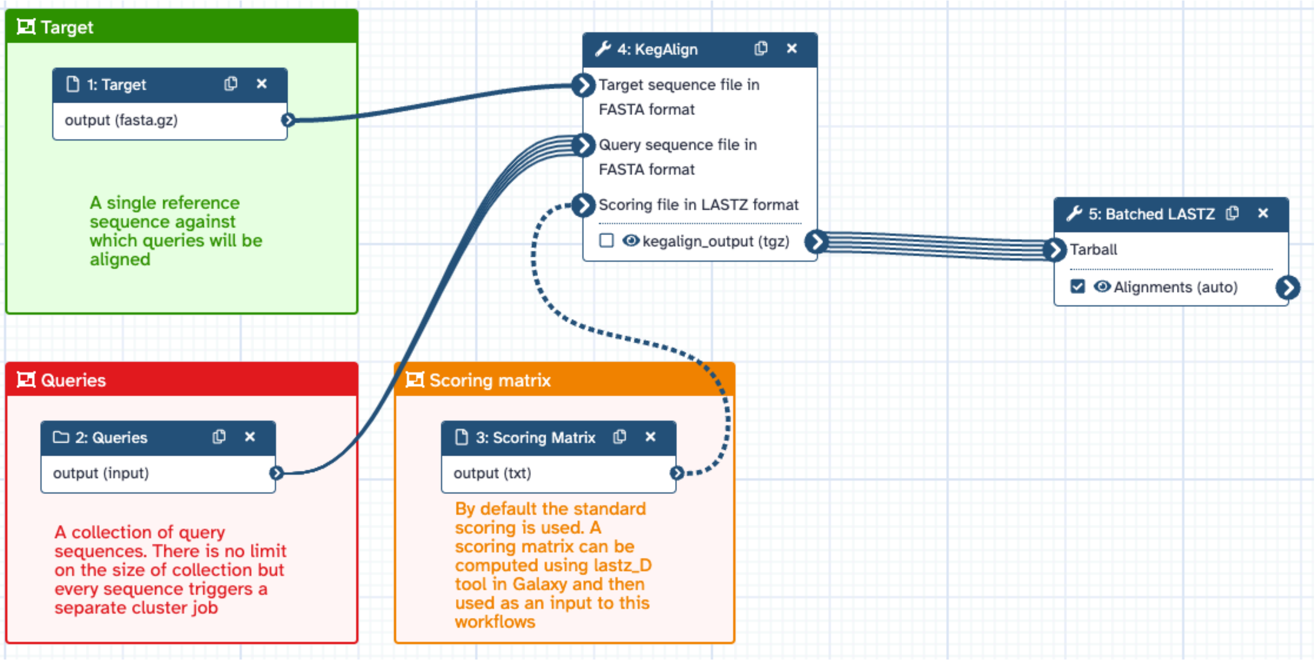

Supplemental Figure 2. KegAlign workflow available at https://usegalaxy.org/u/cartman/w/cdsoverlaps
